# Online Health Information Seeking Behavior: A Systematic Review

**DOI:** 10.3390/healthcare9121740

**Published:** 2021-12-16

**Authors:** Xiaoyun Jia, Yan Pang, Liangni Sally Liu

**Affiliations:** 1 Business School, Foshan University, Foshan 528225, China; Dr.SophiaJia@outlook.com; 2 Massey University, Auckland 0632, New Zealand; 3 Hunan Key Laboratory of Intelligent Logistics Technology, Central South University of Forestry and Technology, Changsha 410004, China; 4 School of Humanities, Media and Creative Communication, Massey University, Auckland 0632, New Zealand; L.Liu2@massey.ac.nz

**Keywords:** online health information seeking behavior, online HISB, health information consumers, social

## Abstract

The last five years have seen a leap in the development of information technology and social media. Seeking health information online has become popular. It has been widely accepted that online health information seeking behavior has a positive impact on health information consumers. Due to its importance, online health information seeking behavior has been investigated from different aspects. However, there is lacking a systematic review that can integrate the findings of the most recent research work in online health information seeking, and provide guidance to governments, health organizations, and social media platforms on how to support and promote this seeking behavior, and improve the services of online health information access and provision. We therefore conduct this systematic review. The Google Scholar database was searched for existing research on online health information seeking behavior between 2016 and 2021 to obtain the most recent findings. Within the 97 papers searched, 20 met our inclusion criteria. Through a systematic review, this paper identifies general behavioral patterns, and influencing factors such as age, gender, income, employment status, literacy (or education) level, country of origin and places of residence, and caregiving role. Facilitators (i.e., the existence of online communities, the privacy feature, real-time interaction, and archived health information format), and barriers (i.e., low health literacy, limited accessibility and information retrieval skills, low reliable, deficient and elusive health information, platform censorship, and lack of misinformation checks) to online health information seeking behavior are also discovered.

## 1. Background

With the proliferation of information and communication technology, utilizing the Internet to seek health information becomes a prevalent behavior [1,2,3,4]). Recently, seeking health information online has become a preferred way due to its availability and coverage of information, the convenience of searching, affordability of access, interactivity and anonymity [5,6,7,8]. Online health information consumers can be patients and their families/friends, and people who purposely seek health-related information online to pursue good health or lifestyle [9,10]. Health information sought online includes “anything regarding the symptoms, diagnoses, and treatments of different diseases or simply general information about weight loss, healthy diets or wellness tips” [11]. Seeking health information online allows health information consumers to obtain knowledge about their health issues, deal with health problems, and make health decisions, and have behavior change [11,12].

Online health information seeking behavior (HISB) has become a global trend [13]. There has been a continuous increase in online health information seeking activities lately [6]. For example, a recent survey conducted in 2020 shows that generally, 55% of Europeans aged 16–74 have sought health-related information online, with a 21% increase since 2010 [14]. In particular, the percentage of online health information seeking has reached over 70% in Finland, Netherlands, Denmark, and Germany [14]. The situation in the US is similar. A recent study indicated that in 2008, 61.2% of the population sought health information online first for their most recent search, while in 2017, the percentage had reached 74.4% [15]. In some Asian countries, the proportion of online health information seeking behavior is even higher. For example, the percentages in mainland China, Philippines, Hong Kong, Indonesia and Vietnam are 79%, 80%, 85%, 85% and 86%, respectively [6,16].

Online health information seeking behavior is thought to have a positive influence on health information consumers since they are more likely to better adhere to treatment after obtaining adequate information of their health conditions [4,5,17]. Hence, understanding online HISB is becoming increasingly important. Online HISB has been changing with the development of information technology and social media, indicating that there might exist significant differences between the health information of consumers’ previous online HISB and the current one. Thus, it is essential to review the most recent academic papers to have an overall up-to-date picture of online HISB research, provide timely and useful guidance to governments, health organizations, media, and online platforms to support and promote online HISB, and direct future studies.

We therefore conduct a systematic review on journal papers and top conference papers published since 2016. The reasons we only review articles published in the last 5 years are twofold. Firstly, it can make sure the findings of online HISB are up-to-date and instructive; secondly, it can supplement the relevant findings of online HISB which reviewed articles published before 2016 [18,19,20]. We ran a search in Google Scholar and chose journal articles and top conference papers with an aim or a result about online HISB. Details of our selection criteria are stated in Section 2.1. Among all the searched articles, after carefully reading their titles and abstracts, we finally included and analyzed 20 articles in our review.

This review intends to integrate the most recent findings of research on online HISB. In detail, the objectives of our review include (i) to discover the general characteristics of online HISB; (ii) to identify factors that could influence online HISB; (iii) to find out the facilitators and barriers to online HISB; (iv) to provide suggestions to health agencies/organizations and social media platforms to support and promote online HISB, and improve their services of online health information provision/access; and (v) to discuss the limitations of current research and come up with suggestions for future studies and future research directions.

This review is organized as follows. Section 2 details the methods, including the search and selection strategy. In Section 3, we present our integrated findings, and in Section 4, we discuss the articles we reviewed and direct future studies.

## 2. Methods

### 2.1. Search Strategy

An electronic search was searched in August 2021 in Google Scholar with the combination of the following terms: “online” AND “health information seek*”. The asterisk (*) was used to account for different variant forms such as “health information seeking” or “health information seeker”. We decided to review papers published in the last five years (since 2016) as social media has had a swift development in recent years. On the one hand, only involving papers published recently ensures that the findings integrated into our systematic review are up to date; on the other hand, it could avoid repetitive review work on online HISB [18,20].

### 2.2. Inclusion and Exclusion Criteria

Full published papers which investigate health information consumers’ online HISB were sought. The relevance was evaluated with the following criteria:

1. The article should (at least partially) have an aim or a result regarding health information seeking;
2. The HISB should take place in an online environment. For example, the social media platform.

The articles were excluded if they were satisfied any one of the following criteria:

1. The article focuses on the evaluation of the algorithm, search engines, or information technology to improve online health information seeking;
2. The article is not written in English;
3. The article is not published in the peer-reviewed journals or top proceedings, such as chapters or books;
4. The article is a review.

**Figure 1:**
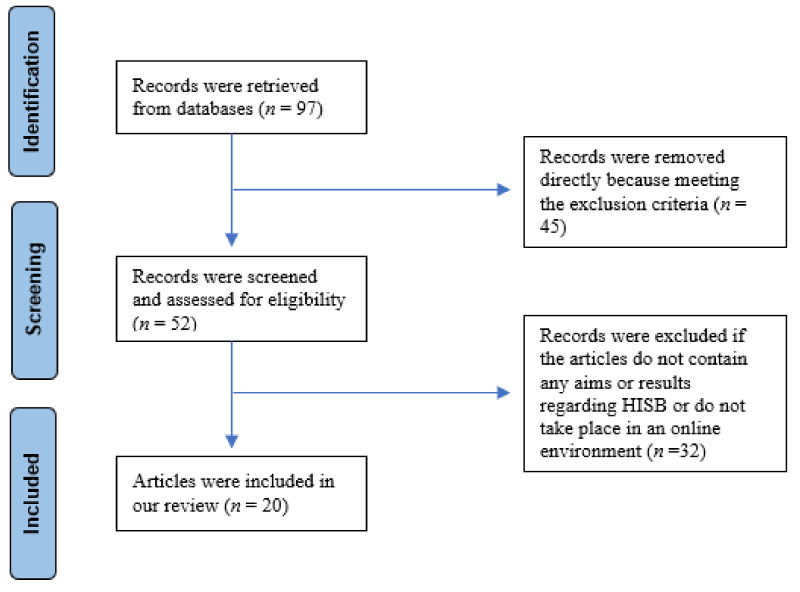
The flowchart of records retrieved, removed, and included.

The selection process is shown in Figure 1.

### 2.3. Selected Literature

The titles and abstracts of the searched articles were carefully read by investigators. Based on the selection criteria as presented in Section 2.2, among 97 articles from the search results, 20 final articles were confirmed to meet the selection criteria, while 77 articles were eliminated from the review because 2 of them are review papers, 9 of them are not written in English, 3 of them are about technology, system evaluation or algorithm, and the rest of the papers are not related to online health information seeking behavior (HISB) or were not published in the peer-reviewed journals or top proceedings.

## 3. Results

After thoroughly analyzing all the articles reviewed, we clustered and categorized the findings based on themes. We presented our findings according to themes extracted from the clustering analysis.

### 3.1. General Profile of the Reviewed Articles

**Figure 2:**
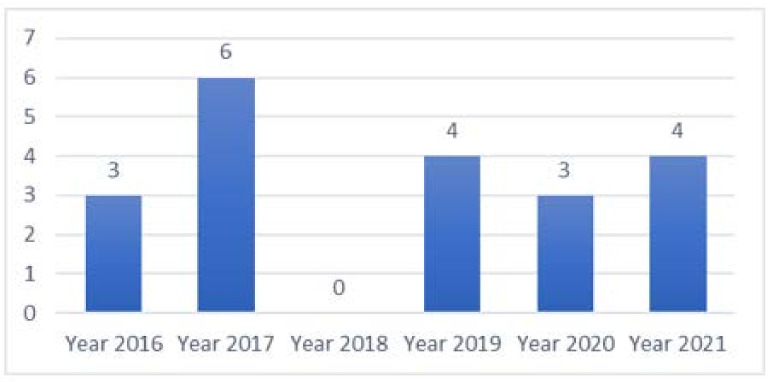
The number of reviewed papers since 2016.

Figure 2 depicts the number of final reviewed papers in online HISB published each year since 2016. Table 1 summarizes the details of the articles reviewed, including research design, methods used, sample sizes, targeted locations, and targeted subjects.

### 3.2. The Identified Characteristics and Influencing Factors of Online Health Information Seeking Behavior

#### 3.2.1. General Behavioral Patterns of Online Health Information Seeking

Online health information seeking has been explored from different perspectives. Overall, according to the reviewed literature, a large majority of the health information consumers thought online health information was important, and online HISB had a positive influence on their health [4,5]. Seventy-five percent of the health information consumers believed the obtained online health information had either a minor or major impact on them (or their families and friends) in their health treatment decision making, overall health maintaining approach, and the way the health information consumers thought about health-related issues [4].

Regarding the frequency of online HISB, over 60% of the health information consumers sought at least on a weekly basis [4]. In particular, more than a quarter of the health information consumers even searched online health information several times a day [4]. It is also found that the frequency of Internet use could affect online HISB. Normally, regular Internet consumers tended to have the habit of seeking health information online [11,25]. Additionally, smartphone users were more likely to search health-related information online than regular cell phone users [11].

According to our review, 83% of the health information consumers searched health information using a general search engine, such as Google and Yahoo, while 15% of the online seekers tended to search on specific health information websites [4]. Social media were more likely to be searched and used to address stress-related issues [21]. When it comes to online health information seeking sources, health information consumers mentioned social media platforms, user-generated online information sites such as Quora and Reddit, and specialized health information websites, etc. [24].

As for the criteria for health websites, what the health information consumers valued the most included (i) accuracy, (ii) currency of information, and (iii) ease of understanding [4]. These selection criteria are consistent with those findings in a similar study [5]. Other criteria the health information consumers thought highly of, including “comprehensiveness, readability, confidentiality, interactivity, and quality of links to use of multimedia and appearances,” [5].

The topics the health information consumers searched online are various. The most popular searched topics included diet and nutrition, exercise and fitness, certain health diseases, specific health treatment, mental health issues, and sexual/productive health information, etc. [4,34].

As for the accessibility of online health information, most online health information consumers believed they could find the information they wanted in their online searching, and 78% of them reported their success rate of online health information seeking was over 60% [4]. As for the reliability of the online health information sources, only 40% of the surveyed health information consumers believed the online health information was reliable [34]. So far, there have been no gender differences found regarding the perception of accuracy of online health information sought [34]. Overall, more than 60% of the health information consumers searched at least three different websites to locate and track health information [4]. Interestingly, still, half of the health information consumers would consult and confirm the health information they found online with health professionals [4].

Our systematic review shows that the reasons for seeking online health information are diverse. Most health information consumers agreed that searching health information online was convenient, anonymous and confidential, time-efficient, and that it was easy to obtain instant up-to-date related information, etc. [4,5]. In addition, searching health information online could give health information consumers the opportunities to seek out sensitive health topics which they felt were hard to talk about in their daily life, obtain the diagnosis of new health-related problems, and access comprehensive information to make health decisions [4].

#### 3.2.2. The Impact of Social and Demographic Factors on Online Health Information Seeking Behavior

Generally age, gender, income, and employment status were found to significantly influence online HISB [11,17,25,27]. According to our review, more women than men were found to seek online health information [4,17,25]. Also, young adults were more likely to seek online health information than senior ones [4,11,25]. There has been only one contradictory finding showing that age was not significantly associated with online HISB [17]. Regarding income, health information consumers with high income were more likely to be online health information seekers [11]. Although employment status has been confirmed to be an influencing factor, current research has not yet indicated how it impacts online HISB [27].

In addition, literacy (or education) levels were believed to affect online HISB [17,25,27]. Health information consumers with a higher health literacy (or education) level reported a higher percentage of online health information seeking. For example, in Ghana, nearly 70% of the surveyed university students sought health information online and interacted with health experts via social media platforms [5]. In Oman, the percentage of college students’ online HISB reached 89% [32]. Both data were significantly higher than those of the general adults as mentioned above. Similarly, in Turkey, health information consumers with higher literacy/education levels were found to seek online health information more frequently than those with lower literacy (or education) levels [25]. Besides, health information consumers with higher health literacy reported fewer difficulties (e.g., fewer effort to get the information, less frustration with health information search, less concern about health information quality, and fewer difficulties in understanding health information) with online health information search than those with lower health literacy [31]. Moreover, health information seeking sources also have an impact on health literacy. The existing research shows that health information consumers who sought health information from the Internet were found to have a higher health literacy level than those who sought health information from other traditional sources such as newspapers and television [23].

Most studies confirmed that country of origin and place of residence also had an impact on online HISB [25]. There were more online health information seekers in developed countries (or regions) than those in developing countries (or regions). For example, in America, 63% of the surveyed adults reported they had online HISB [26]; in Turkey, the percentage of online HISB was 48.6% [23] while in Ghana, this percentage was only 31.4% [17]. Another study conducted in Turkey also indicates that health information consumers from developed regions sought online health information more frequently than those from less-developed regions [25].

The caregiving role can also influence online HISB. Caregivers, compared with the general public, were more likely to seek health-related information online [33]. Online HISB among caregivers could even be used to predict the health of the family [33]. However, some other social and demographic factors such as religion, marital status, and parental status were not found to affect online HISB [17].

Furthermore, social and demographic characteristics were believed to be associated with the searched health website type [30]. Young health information consumers were found to prefer seeking health information from commercial websites, while senior health information consumers tended to search from academic websites. In terms of gender, women were more likely to seek health-related information from government websites. Health information consumers with lower income were more likely to seek information using the general search engines. Regarding ethnicity, Hispanic health information consumers were more likely to seek health information using a general search engine compared with their white counterparts [30].

#### 3.2.3. Other Variables and Online Health Information Seeking Behavior

According to our review, factors initiating online HISB have been surveyed. The top three factors were personal, family, and friend/peer sickness [32]. In addition, there were some factors that could influence online HISB, including currency of information, ease of understanding of health-related information, the accuracy of the information, and content readability, etc. [5]. Prior research has also proposed different models to explore the antecedents of online HISB. Four relevant models have been identified as follows:

Comprehensive model of information seeking (CMIS) has been widely used to investigate factors determining individuals’ information seeking behavior [36,37,38]. Bernadas and Jiang [28] adopted CMIS and tested it in the online health context. They validated that trustworthiness and tailorability of channels could positively influence online HISB. Here, tailorability means “the degree to which a source makes it possible to acquire information that is unique to one’s circumstance or situation” [39].

**Figure 3:**
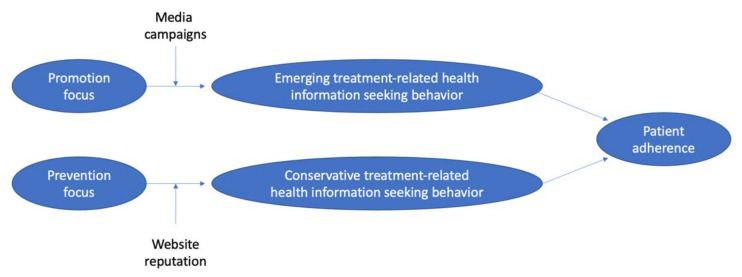
The research model of Zhang et al. [29]. In this model, health information consumers with different focuses were found to seek different online health information.

Based on regulatory focus theory, which suggests that health information consumers with different regulatory focuses normally behave in different ways, Zhang et al. [29] believe health information consumers in different focus states would seek different online health information. They proposed a research model (as shown in Figure 3) and tested it in health information consumers with two different focuses—promotion focus and prevention focus [29]. Promotion focus was related to advancement, growth, and an aim of achieving progress; while prevention focus was linked to security, safety, and an aim of avoidance of mismatches [29]. They confirmed that: (1) online health information consumers with promotion focus tended to have emerging treatment-related HISB; (2) those with prevention focus tended to follow routines, and therefore have conservative treatment-related HISB; (3) with the increase of media campaigns, the association between promotion focus and emerging treatment-related HISB would be strengthened; (4) with the increase of website reputation, the association between prevention focus and conservative treatment-related HISB would be strengthened. Besides, patients’ conservative treatment-related HISB with a prevention focus was validated to positively influence their adherence [29]. This finding is similar to that of another study [35], indicating that online health information seeking correlated with health (cancer) screening adherence. In general, people who sought health-related information online were more likely to get screened on schedule [35]. In comparison, emerging treatment-related HISB with a promotion focus was found to have no relationship with health information consumers’ adherence [29].

**Figure 4:**
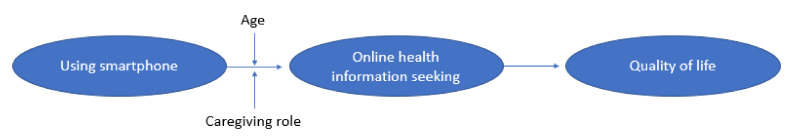
The partial research model of Ghahramani and Wang [11]. In this model, online health information seeking behavior was found to mediate the relationship between smartphone use and quality of life.

Ghahramani and Wang [11] developed a research model exploring the impact of smartphone uses on the quality of life from the perspective of online health information behavior. Both health information seeking and tracking behavior was involved in the proposed model. As we only review seeking behavior, the partial related model is relevant and shown in Figure 4. Ghahramani and Wang [11] verified that the use of smartphones had a positive effect on HISB. Moreover, age and caregiving roles could mediate the association between smartphone use and HISB [11]. Young smartphone users and caregivers who were smartphone users were more likely to seek health information online than senior and non-caregivers smartphone users [11]. In addition, online HISB was confirmed to positively affect the quality of life [11]. Online HISB could help health information consumers obtain health knowledge, deal with health conditions, reduce pressure and stress caused because of health problems, and therefore have a better life [11].

**Figure 5:**
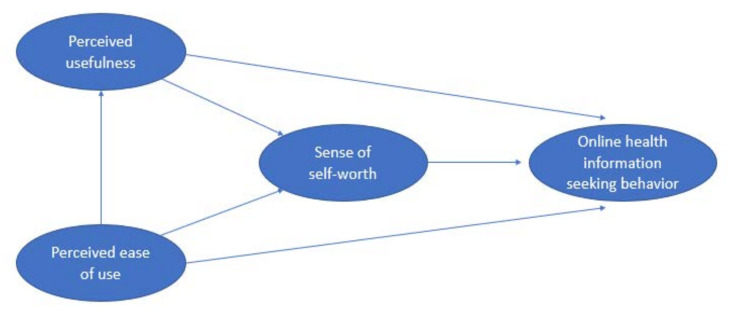
The research model of Boyce et al. [22]. In this model, online health information seeking behavior was found to be influenced by perceived usefulness, perceived ease of use and sense of self-worth.

Boyce et al. [22] also proposed a research model (as shown in Figure 5) to explore the factors influencing online HISB. They validated that perceived usefulness had a positive influence on HISB, which is consistent with the findings of Ghahramani and Wang [11]. In addition, perceived usefulness could mediate the relationship between perceived ease of use and online HISB. Besides, the sense of self-worth was also confirmed to mediate the relationship between perceived ease of use and online HISB.

### 3.3. Facilitators and Barriers to Online Health Information Seeking

Facilitators of online health information seeking have been discussed by researchers. First of all, online communities allow health information seekers to have a connection and share information with specific health information consumers. Second, the privacy feature online makes health information consumers feel safe to seek information. Third, the synchronous function online allows health information consumers to interact in real-time through online dialogue. By posting questions and receiving feedback, health information consumers could obtain immediate health information required and make use of it. Additionally, online health information is always available in an archival format. Hence, health information consumers can obtain enough information and access the knowledge even posted in the past [24].

Barriers to seeking online health information in existing research have been analyzed from consumer factors, health information factors, and platform factors [9,24]. Regarding consumer factors, firstly, people with low health literacy may not even realize their need to seek online health information. Secondly, health information consumers who are not able to access the Internet, or with limited information retrieval skills may negatively affect their online health information seeking as well [9].

In terms of health information factors, poor quality and low reliability of the online health information (abundant misinformation) hinder health information consumers’ trust, and therefore negatively influence their online seeking behavior [9,24]. In addition, the content of online health information is not adequate to meet health information consumers’ demands. Some health-related information is still lacking, such as the knowledge of prevention, and psychological and emotional guidance [9]. Furthermore, with the explosively growing online health-related information, it is difficult for health information consumers to quickly capture the information they require. In particular, some online health information with medical jargon is hard for health information consumers to understand [9].

When it comes to platform factors, firstly, the platform censorship of the content and identity makes health information consumers inconvenient to join online communities where information is shared and exchanged between online members [24]. In addition, lacking mechanisms of misinformation checks make it hard for health information consumers to distinguish credible information from incorrect knowledge [24]. These two factors were thought to have negatively affected online HISB [24].

### 3.4. Suggestions to Platforms, Health Organizations, and Governments

There are quite a few research papers which focus on providing suggestions to improve online health information platforms, and services from governments and health organizations. Thematically, we grouped these suggestions into three categories: (i) design suggestions to online social media platforms, (ii) suggestions to health-related workers and professionals, and (iii) suggestions to relevant health organizations and governments.

*Design suggestions for social media platforms.* Health information consumers’ ideal platform has been investigated. Health information consumers indicated that their ideal social media platform should be cleanly designed, easily navigated, and seem similar to a blend of current existing platforms (e.g., YouTube and Twitter) with greater inclusivity and accessibility for diverse identities and experiences [24]. Health information consumers also mentioned some features they like: (1) platforms are expected to include a discussion page where health information consumers could ask health-related questions and receive answers from healthcare experts; (2) platforms should allow health information consumers to set appropriate identity labels by providing more options. For example, allowing health information consumers to have the opportunities to choose other sex types rather than the binary choices of male and female; (3) platforms should emphasize privacy and security; (4) platforms should consider content moderation balance to minimize false information and encourage useful information exchange [24].

*Suggestions to health-related workers and professionals.* Health professionals can be actively involved in motivating, assisting, and teaching their health information consumers about health information seeking online [17]. For example, health experts can recommend “provider-approved” online resources to the general public [30]). Website editors also play an important role in disseminating health information online. They are supposed to consult health experts to make sure the health-related information is accurate, readable, and understandable before they publish it online [30].

*Suggestions to governments and health organizations.* Firstly, governments and health organizations are expected to raise health awareness of the public. When the public are aware of the importance of health, they are more likely to actively take part in their health management [9].

Secondly, governments and health organizations are expected to design and improve the construction of responsive websites/platforms and applications to increase the accessibility of online healthcare and promote e-health [5,9]. An e-library is also expected to be built to provide free and reliable online health-related information to the public at any time [9].

Thirdly, online websites, platforms, and mobile healthcare applications should be encouraged to use to improve health outcomes. Subsidies and other incentives from governments and health organizations may be considered to promote their adoption [33].

In addition, online health-related information needs to be monitored, reviewed, and assessed to ensure its accuracy, completeness, and consistency [25,33]. Governments and health organizations may establish authoritative evaluation platforms to guarantee the quality of the information [9]. Online health information also needs to be delivered through proper media channels and platforms to fit health information consumers’ media utility patterns [28].

Last but not least, more strategies need to be developed and evaluated to make online health information more accessible and understandable, and make online health information seeking easier [31]. In particular, health literacy needs to be taken into account when governments and health organizations plan information seeking and dissemination strategies [31]. The evolving technologies continuously generate new ways and channels for health information delivery. Hence, health information consumers may have to constantly learn new skills [31]. Governments and health organizations are expected to provide training and support to improve health information consumers’ health literacy, especially those with low health literacy [9,25]. In addition, more up-to-date research regarding new strategies to improve health literacy, and health information dissemination and seeking should be encouraged as well [31].

## 4. Discussion, Limitations, and Future Work

With the rapid growth of information technology and social media, online HISB has been changing and evolving with time. In other words, the current online HISB of health information consumers may be hugely different from that of ten years ago. Reviewing articles published in the last five years ensures the findings of online HISB are up-to-date, and therefore can provide timely and meaningful reference to governments, health organizations, and media platforms.

Overall, most related findings on online HIBS published in the last five years are consistent. This systematic literature review with integrated findings offers us a whole picture regarding the general characteristics of online HISB, factors affecting online HISB, and what governments, health organizations, and social media platforms should do to provide better services to health information consumers. Methodologically, a majority of the studies reviewed are quantitative and were conducted using a survey with a self-report style [5,11,21,22,23,24,25,26,27,28,29,30,31,32,33,35]. Data collected through self-report may not represent the genuine situation, thinking, or behavior of some health information consumers. Future studies may consider adopting or triangulating other methods (e.g., observations) to counteract the disadvantages of the method used [40,41] when investigating online HISB. Some data, such as consumers’ online searching records, can be included to analyze online HISB as well.

From the perspective of the subjects, although the articles reviewed cover a wide range of population, some specific groups of the population are lacking. The subjects of most studies reviewed are general adults [4,11,24,25,30,31,35]. Participants also include university students [5,32], trans or nonbinary adults [24], foreign workers [28], reproductive-age women [21] and mothers [27], and patients with chronic disease [22]. Still, some specific and relevant groups of the population have not been involved in online HISB, such as children and adolescents, mental health patients, teachers, health educators, health workers and professionals, and disabled users. Hence, future studies are expected to investigate online HISB in different groups of subjects, and compare their online HISB.

The targeted locations of the studies reviewed include various countries including America, China, Turkey, Ghana, Malaysia, Oman, Korea, and Nigeria, however there is still a lack in a wide range of countries such as European countries and Latin American countries. There is also a lack of comparison studies among countries with a similar economic development level (e.g., American health consumers’ online HISB vs. Australian health consumers’ online HISB), and among places with similar cultural backgrounds (e.g., online health consumers’ information seeking behavior in mainland China vs. that in Hong Kong). Future studies could conduct cross-country studies and compare behavioral similarities and differences. As for the content, most studies reviewed only focus on investigating and reporting the very basic characteristics of online HISB without providing deeper insights. For example, some studies only surveyed the percentage of online HISB [23,26,30,34], only providing the general and superficial understanding of online HISB. Future studies are expected to design comprehensively and investigate online HISB in depth from different aspects. Studies on facilitators and barriers are still limited and need to be investigated further. Exploring facilitators and barriers is helpful to maintaining the good aspects which are valued by health consumers, and to discover problems. Moreover, understanding facilitators and barriers could help to come up with suggestions to governments, health organizations, and health platforms. In particular, more strategies should be explored, and detailed suggestions (e.g., technical side) need to be provided in future studies. On the other hand, governments, health organizations, and health platforms should refer to these strategies and create practical solutions to generate an open, safe and vibrant online environment, make sure the online health information is correct and precise, and make health information seeking easier, more accessible and efficient. For governments, health organizations, and health platforms, the effective suggested solutions put forward based on empirical studies should be applied in practice rather than only kept as suggestions in the paper. This review is not without limitations. First of all, the search strategy may miss out some related studies which were not identified in Google Scholar by searching the keywords. Second, our standards of eligible articles may be different from others. Hence, the findings may be different.

## Figures and Tables

**Table 1:** The details of the articles reviewed.

Authors	Publication Year	Title	Journal	Research Design	Data Collection Method	Sample Number	Subjects (Age Group/Sex/etc.)	Location	Data Analysis
Ukonu & Ajaebili, 2021 [21]	2021	Socio-cultural determinants of women’s health information opportunities in Nsukka, southeast Nigeria	Asian Women	Quantitative	Survey	*n* = 591	Reproductive-age women	America	Descriptive and correlation analysis
Boyce et al., 2021 [22]	2021	Exploring the factors in information seeking behavior: a perspective from multinational COPD online forums	Health Promotion International	Quantitative	Survey	*n* = 201	Participants with chronic obstructive pulmonary disease (COPD).	America & other countries such as New Zealand, Australia, South Africa, the UK, Philippines, etc.	PLS-SEM (partial least squares structural equation modeling)
Özkan et al., 2021 [23]	2021	The relationship between health literacy level and media used as a source of health-related information	HLRP: Health Literacy Research and Practice	Quantitative	Survey	*n* = 6228	Turkish citizen	Turkey	Descriptive and linear regression analysis
Augustaitis et al., 2021 [24]	2021	Online transgender health information seeking: facilitators, barriers and future directions	Proceedings of the 2021 CHI Conference on Human Factors in Computing Systems	Qualitative	Online focus groups	*n* = 26	Trans and/or nonbinary adults	America	Inductive open coding approach
Demirci et al., 2021 [25]	2021	Socio-demographic characteristics affect health information seeking on the Internet in Turkey	Health Information & Libraries Journal.	Quantitative	Survey	*n* = 19,389	Turkish	Turkey	Logistic regression
Ghahramani & Wang, 2020 [11]	2020	Impact of smartphones on quality of life: a health information behavior perspective	Information Systems Frontiers	Quantitative	Survey	*n* = 3014	American adults	America	Pearson’s correlation analysis, Preacher and Hayes Process macro
Lee et al., 2020 [26]	2020	Communication about health information technology use between patients and providers	Journal of General Internal Medicine	Quantitative	Survey	*n* = 970	Adult residents in 34 Indiana counties with higher cancer mortality rates than the state average	America	Descriptive statistics and multivariable logistic regression
Lee, 2020 [27]	2020	Which individual characteristics influence mothers’ health information-seeking behavior?	Journal of the Korean Society for Library and Information Science	Quantitative	Survey	*n* = 851	Among mothers of healthy infants and toddlers (currently living in the US or Korea)	America & Korea	Ordinal regression analyzes
Bernadas & Jiang, 2019 [28]	2019	Explaining online health information seeking of foreign domestic workers: a test of the comprehensive model of information seeking	Health and Technology	Quantitative	Survey	*n* = 300	Filipino female foreign domestic workers (WFDs) in HK	Hong Kong	Multiple OLS regression
Nangsangna & Vroom, 2019 [17]	2019	Factors influencing online health information seeking behavior among patients in Kwahu West Municipal, Nkawkaw, Ghana	Online Journal of Public Health Informatics	Quantitative	Survey	*n* = 204	Ghanaian residents who are over 16	Ghana	Descriptive statistics, Chi-square test, and logistic regression
Zhang et al., 2019 [29]	2019	Why do patients follow physicians’ advice? The influence of patients’ regulatory focus on adherence: An empirical study in China	BMC Health Services Research	Quantitative	Survey	*n* = 336	Chinese patients with health information seeking experience and hospital treatment experience	China	Structural equation modeling and confirmatory factor analysis
LaValley et al., 2017 [30]	2017	Where people look for online health information	Health Information & Libraries Journal	Quantitative	Survey	*n* = 3959	American adults	America	Regression analysis
Maon et al., 2017 [4]	2017	Online health information seeking behavior pattern	Advanced Science Letters	Quantitative	Survey	*n* = 482	Malaysian adults	Malaysia	Descriptive analysis
Osei Asibey et al., 2017 [5]	2017	The Internet use for health information seeking among Ghanaian university students: A cross-sectional study	International Journal of Telemedicine and Applications	Quantitative	Survey	*n* = 650	Ghanaian university students	Ghana	Descriptive analysis
Manganello et al., 2017 [31]	2017	The relationship of health literacy with use of digital technology for health information: implications for public health practice	Journal of Public Health Management and Practice	Quantitative	Survey	*n* = 1350	New York State adult residents	America	A weighted analysis
Sultan et al., 2017 [32]	2017	Health information seeking behavior of college students in the sultanate of Oman	Khyber Medical University Journal	Quantitative	Survey	*n* = 200	Omani college students (adults)	Oman	Descriptive statistics and chi-square tests
Kim et al., 2017 [33]	2017	Seeking medical information using mobile Apps and the Internet: Are family caregivers different from the general public?	Journal of Medical Systems	Quantitative	Survey	*n* = 3677 [*n* = 2425 (family caregivers) *n* = 1252 (Non-family caregivers)]	American adults	America	Multivariate logistic regression
Xuexia et al., 2016 [9]	2016	Analysis of barriers to health information seeking and utilizing in patients with diabetes	Cross-Cultural Communication	N/A	N/A	N/A	N/A	China	N/A
Obasola & Agunbiade, 2016 [34]	2016	Online health information seeking pattern among undergraduates in a Nigerian University	SAGE Open	Quantitative	Survey	*n* = 400	Nigerian undergraduate students	Nigeria	Descriptive analysis
Shneyderman et al., 2016 [35]	2016	Health information seeking and cancer screening adherence rates	Journal of Cancer Education	Quantitative	Survey	Did not indicate	American adults	America	Descriptive statistics and regression modeling

## Data Availability

Not applicable.

## References

[1] Bundorf M.K., Wagner T.H., Singer S.J., Baker L.C. (2006). Who searches the internet for health information?. Health Serv. Res..

[2] Renahy E., Parizot I., Chauvin P. (2010). Determinants of the frequency of online health information seeking: Results of a web-based survey conducted in France in 2007. Inform. Health Soc. Care.

[3] Lee Y.J., Boden-Albala B., Larson E., Wilcox A., Bakken S. (2014). Online health information seeking behaviors of Hispanics in New York City: A community-based cross-sectional study. J. Med. Internet Res..

[4] Maon S.N., Hassan N.M., Seman S.A.A. (2017). Online health information seeking behavior pattern. Adv. Sci. Lett..

[5] Osei Asibey B., Agyemang S., Boakye Dankwah A. (2017). The Internet use for health information seeking among Ghanaian university students: A cross-sectional study. Int. J. Telemed. Appl..

[6] Chu J.T., Wang M.P., Shen C., Lam T.H., Viswanath K., Chan S.S.C. (2017). How, when and why people seek health information online: Qualitative study in Hong Kong. Interact. J. Med. Res..

[7] Powell J., Inglis N., Ronnie J., Large S. (2011). The characteristics and motivations of online health information seekers: Cross-sectional survey and qualitative interview study. J. Med. Internet Res..

[8] Lagoe C., Atkin D. (2015). Health anxiety in the digital age: An exploration of psychological determinants of online health information seeking. Comput. Hum. Behav..

[9] Wei X., Du Z., Zhang S. (2016). Analysis of barriers to health information seeking and utilizing in patients with diabetes. Cross-Cult. Commun..

[10] Lu L., Liu J., Yuan Y.C. (2020). Health Information Seeking Behaviors and Source Preferences between Chinese and US Populations. J. Health Commun..

[11] Ghahramani F., Wang J. (2020). Impact of smartphones on quality of life: A health information behavior perspective. Inf. Syst. Front..

[12] Lambert S.D., Loiselle C.G. (2007). Health information—Seeking behavior. Qual. Health Res..

[13] Zhang D., Zhan W., Zheng C., Zhang J., Huang A., Hu S., Ba-Thein W. (2021). Online health information-seeking behaviors and skills of Chinese college students. BMC Public Health.

[14] (2021). Eurostat, One in Two EU Citizens Look for Health Information Online. https://ec.europa.eu/eurostat/web/products-eurostat-news/-/edn-20210406-1.

[15] Finney Rutten L.J., Blake K.D., Greenberg-Worisek A.J., Allen S.V., Moser R.P., Hesse B.W. (2019). Online health information seeking among US adults: Measuring progress toward a healthy people 2020 objective. Public Health Rep..

[16] Wang X., Shi J., Kong H. (2021). Online health information seeking: A review and meta-analysis. Health Commun..

[17] Nangsangna R.D., Vroom F.D.C. (2019). Factors influencing online health information seeking behavior among patients in Kwahu West Municipal, Nkawkaw, Ghana. Online J. Public Health Inform..

[18] Zhao Y., Zhang J. (2017). Consumer health information seeking in social media: A literature review. Health Inf. Libr. J..

[19] Tan S.S.L., Goonawardene N. (2017). Internet health information seeking and the patient-physician relationship: A systematic review. J. Med. Internet Res..

[20] Marton C., Choo C.W. (2012). A review of theoretical models of health information seeking on the web. J. Doc..

[21] Ukonu M.O., Ajaebili N.C. (2021). Socio-cultural determinants of women’s health information opportunities in Nsukka, Southeast Nigeria. Asian Women.

[22] Boyce L., Harun A., Prybutok G., Prybutok V.R. (2021). Exploring the factors in information seeking behavior: A perspective from multinational COPD online forums. Health Promot. Int..

[23] Özkan S., Tüzün H., Dikmen A.U., Aksakal N.B., Çalışkan D., Taşçı Ö., Güneş S.C. (2021). The relationship between health literacy level and media used as a source of health-related information. HLRP.

[24] Augustaitis L., Merrill L.A., Gamarel K.E., Haimson O.L. Online transgender health information seeking: Facilitators, barriers, and future directions. Proceedings of the 2021 CHI Conference on Human Factors in Computing Systems.

[25] Demirci Ş., Uğurluoğlu Ö., Konca M., Çakmak C. (2021). Socio-demographic characteristics affect health information seeking on the Internet in Turkey. Health Inf. Libr. J..

[26] Lee J.L., Rawl S.M., Dickinson S., Teal E., Baker L.B., Lyu C., Tarver W.L., Haggstrom D.A. (2020). Communication about health information technology use between patients and providers. J. Gen. Intern. Med..

[27] Lee H.S. (2020). Which individual characteristics influence mothers’ health information-seeking behavior?. J. Korean Soc. Libr. Inf. Sci..

[28] Bernadas J.M.A.C., Jiang L.C. (2019). Explaining online health information seeking of foreign domestic workers: A test of the comprehensive model of information seeking. Health Technol..

[29] Zhang R., Lu X., Wu W., Shang X. (2019). Why do patients follow physicians’ advice? The influence of patients’ regulatory focus on adherence: An empirical study in China. BMC Health Serv. Res..

[30] LaValley S.A., Kiviniemi M.T., Gage-Bouchard E.A. (2017). Where people look for online health information. Health Inf. Libr. J..

[31] Manganello J., Gerstner G., Pergolino K., Graham Y., Falisi A., Strogatz D. (2017). The relationship of health literacy with use of digital technology for health information: Implications for public health practice. J. Public Health Manag. Pract..

[32] Sultan K., Joshua V.R., Misra U. (2017). Health information seeking behavior of college students in the Sultanate of Oman. Khyber Med. Univ. J..

[33] Kim H., Powell M.P., Bhuyan S.S. (2017). Seeking medical information using mobile apps and the internet: Are family caregivers different from the general public?. J. Med. Syst..

[34] Obasola O.I., Agunbiade O.M. (2016). Online health information seeking pattern among undergraduates in a Nigerian university. SAGE Open.

[35] Shneyderman Y., Rutten L.J.F., Arheart K.L., Byrne M.M., Kornfeld J., Schwartz S.J. (2016). Health information seeking and cancer screening adherence rates. J. Cancer Educ..

[36] Johnson J.D., Meischke H. (1993). A comprehensive model of cancer-related information seeking applied to magazines. Hum. Commun. Res..

[37] Johnson J.D., Donohue W.A., Atkin C.K., Johnson S. (1995). A comprehensive model of information seeking: Tests focusing on a technical organization. Sci. Commun..

[38] Longo D.R. (2005). Understanding health information, communication, and information seeking of patients and consumers: A comprehensive and integrated model. Health Expect..

[39] Rains S.A., Ruppel E.K. (2016). Channel complementarity theory and the health information-seeking process: Further investigating the implications of source characteristic complementarity. Commun. Res..

[40] Jia X., Wang R., Liu J.H., Xie T., Nah F.F.-H., Siau K. (2020). How to attract more viewers in live streams? A functional evaluation of streamers’ strategies for attraction of viewers. HCI in Business, Government and Organizations.

[41] Jia X., Wang R., Liu J.H., Jiang C. (2021). Discovery of behavioral patterns in online social commerce practice. Wiley Interdiscip. Rev..

